# Hydrophilic Character of Single-Layer MoS_2_ Grown on Ag(111)

**DOI:** 10.1021/acs.jpcc.1c01768

**Published:** 2021-04-27

**Authors:** Francesco Tumino, Carlo Grazianetti, Christian Martella, Marina Ruggeri, Valeria Russo, Andrea Li Bassi, Alessandro Molle, Carlo S. Casari

**Affiliations:** † Department of Energy, Politecnico di Milano, via G. Ponzio 34/3, Milano, I-20133, Italy; ‡ CNR-IMM Unit of Agrate Brianza, via C. Olivetti 2, Agrate Brianza, I-20864, Italy

## Abstract

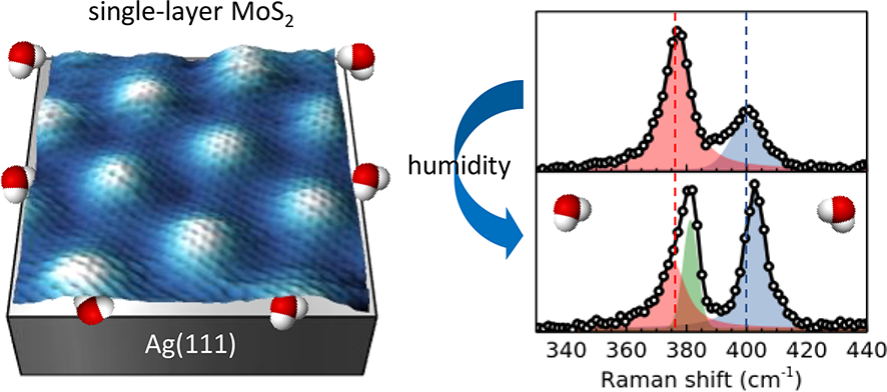

The study of MoS_2_/metal interfaces is crucial for engineering efficient semiconductor–metal contacts in 2D MoS_2_-based devices. Here we investigate a MoS_2_/Ag heterostructure fabricated by growing a single MoS_2_ layer on Ag(111) by pulsed laser deposition under ultrahigh vacuum (UHV) conditions. The surface structure is observed in situ by scanning tunneling microscopy, revealing the hexagonal moiré pattern characteristic of the clean MoS_2_/Ag(111) interface. Ex situ Raman spectroscopy reveals an anomalous behavior of vibrational modes, induced by the strong MoS_2_–Ag interaction. After few-hours exposure to ambient conditions the Raman response significantly changes and the formation of molybdenum oxysulfides is revealed by X-ray photoelectron spectroscopy. These effects are due to the interplay with water vapor and can be reversed by a moderate UHV annealing. A polymeric (PMMA) capping is demonstrated to hinder water-induced modifications, preserving the original interface quality for months.

## Introduction

In the past decade, molybdenum disulfide (MoS_2_) has been extensively studied as a promising 2D material for a wide range of potential applications, such as electronics, optoelectronics, catalysis, and energy storage.^1−3^ Among the many challenges in the route to develop novel MoS_2_-based devices, the fabrication of efficient MoS_2_–metal contacts is one of the most critical. Therefore, the study of MoS_2_–metal heterostructures and their interface properties is necessary to provide a comprehensive understanding of the way MoS_2_ interacts with metals and how such interaction affects its electronic, phononic, and transport properties. It is also crucial to assess the stability of MoS_2_–metal systems by studying their chemical reactivity under ambient conditions, which can have profound effects on device performances.^4−6^

Recent works on MoS_2_–metal heterostructures (mostly MoS_2_–Au) have shown the influence of metallic contacts on electronic, optical, and vibrational properties of MoS_2_.^7−12^ Such an influence is related to metal-induced local strain and charge redistribution, and it is dramatically dependent on the interface properties, especially in terms of purity and morphological homogeneity of the metal contact.^7,13^ The ideal MoS_2_–metal system, serving as model for fundamental studies, should have a contaminant-free, perfectly planar, and atomically sharp interface. However, commonly employed fabrication techniques, like MoS_2_ exfoliation on metal substrates or physical vapor deposition (PVD) of metals on MoS_2_, do not always comply with the above requirements: exfoliation being limited by unavoidable environmental contamination^13^ and PVD by the low metal wettability of MoS_2_, which may lead to nonhomogeneous contact.^7^ An alternative and promising strategy relies on the synthesis of MoS_2_ on a perfectly clean and flat metallic surface under controlled growth conditions, e.g., using ultrahigh vacuum (UHV) compatible techniques. However, synthesizing MoS_2_ on metal substrates with high control on its thickness down to the single-layer (SL) regime is still challenging, as the most widely used chemical vapor deposition (CVD) methods are largely limited to insulating or chemically inert substrates (e.g., SiO_2_).

In this work, we use UHV pulsed laser deposition (PLD) to grow SL MoS_2_ on Ag(111). Our PLD method allows us to finely tune the MoS_2_ coverage in the submonolayer range and to produce uniform SL MoS_2_ films covering the Ag surface on the centimeter-scale. The morphological and structural properties are investigated down to the atomic scale by in situ scanning tunneling microscopy (STM), revealing the characteristic moiré pattern due to the lattice mismatch between MoS_2_ and Ag(111). Raman and photoluminescence (PL) spectroscopy (performed ex situ) provide insight into the film–substrate interplay, revealing profound differences with respect to the well-known vibrational spectrum of SL MoS_2_ supported by silica (or other dielectric substrates) and a complete quenching of the PL signal. The Raman spectrum is also used as fingerprint to monitor the effects of short-term exposure to ambient conditions, revealing a high sensitivity toward humidity. X-ray photoelectron spectroscopy (XPS) provides us with a more complete picture of the chemical behavior of the exposed heterostructure, corroborating the Raman analysis. We finally propose a polymer capping to preserve the original quality of MoS_2_/Ag(111) interface from degradation on the month time-scale, thus addressing a technologically relevant issue in the framework of 2D semiconductor–metal junctions.

## Methods

### Sample Preparation and Exposure

The synthesis and STM characterization of MoS_2_/Ag(111) was carried out in a UHV apparatus (at Politecnico di Milano, base pressure <10^–10^ mbar) equipped with tools for surface preparation, and connected to a dedicated chamber for PLD (base pressure lower than 5 × 10^–9^ mbar). Ag(111)/mica (Mateck) was cleaned in the UHV chamber by cycles of Ar^+^ sputtering (1 keV, 3 × 10^–6^ mbar) and annealing at 700 K. After having checked the Ag surface by STM, we internally transferred the substrate into the PLD chamber. MoS_2_ was deposited at room temperature (RT), using KrF laser pulses (248 nm wavelength, 10 ns pulse duration) to ablate a stoichiometric MoS_2_ target (Testbourne). The pulse energy was set at 200 mJ, yielding a laser fluence on the target of about 2 J/cm^2^. The pulse repetition rate was set at 1 pulse/s, allowing us to easily control the total number of laser pulses. The target–substrate distance was set at 3 cm during depositions. The MoS_2_ coverage was varied by properly tuning the number of laser pulses (between 3 and 15) on the target. After deposition, the sample was annealed at 730 K for 30 min in UHV and then observed by STM at RT. Once taken out from the UHV system, MoS_2_/Ag(111) samples were stored in ambient conditions inside transparent boxes to protect them from dust. Aged samples were restored to their original conditions by annealing them in UHV at 600 K for 2 h. Sample exposure to O_2_, N_2_ and H_2_O was performed in the load-lock chamber of the UHV system (base pressure 5 × 10^–9^ mbar). O_2_ and N_2_ were dosed at ∼1 bar using a needle valve. H_2_O was dosed using an electrically controlled leak valve. The volume of liquid water vaporized in the chamber roughly corresponds to the amount of water vapor in 75% humid air. The exposure time was set to 48 h, enough to induce clearly observable modification in Raman spectra. After Raman measurements, the sample was put back in the UHV chamber, restored by annealing at 600 K for 2 h, and left to cool to room temperature before exposing it to another gas.

### Scanning Tunneling Microscopy

In situ STM measurements were performed at RT using an Omicron microscope. STM images were acquired in constant-current mode using homemade W tips, fabricated by electrochemical etching. Typical measurement parameters were in the range 0.5–2 V for bias voltage and 0.3–0.5 nA for set-point current.

### Raman Spectroscopy

Raman measurements at Politecnico di Milano were performed in backscattering configuration using a Renishaw InVia spectrometer, coupled to an Ar laser. We used a 457 nm (2.71 eV) excitation, a 2400 lines/mm diffraction grating, and a 50× objective lens. The laser power on the sample was kept below 1 mW, taking care to avoid heating effects on the acquired spectra. We calibrated the spectrometer against the 521 cm^–1^ peak of a Si crystal. The acquired spectra were baseline corrected and fitted using Voigt functions. We measured the photoluminescence (PL) signal with the same instrument, using a 514 nm (2.41 eV) excitation and a 1800 lines/mm diffraction grating. Raman measurements at CNR were performed in backscattering configuration employing a Renishaw InVia spectrometer, equipped with the 514 nm (2.41 eV) line of solid-state diode laser and a 2400 lines/mm dispersive grating. The laser radiation was focused on the sample by means of a 50× Leica objective (0.75 numerical aperture), maintaining the incident laser power below 1 mW to avoid sample damage.

### X-ray Photoelectron Spectroscopy

XPS analysis was carried out in a second UHV apparatus (at CNR, base pressure 10^–10^ mbar) by means of in situ nonmonochromatized Mg X-ray source (*hν* = 1253.6 eV) at 37° takeoff angle (surface sensitive). The spectra were decomposed using a product between Gaussian and Lorentzian lineshapes upon Shirley background removal. The energy difference between the S 2p (Mo 3d) spin–orbit doublet was kept equal to 1.172 eV (3.14 eV). The following core levels were recorded before and after UHV annealing and air exposure: S 2p, Mo 3d (plus S 2s), C 1s, Ag 3d, and O 1s.

### Capping

A 9–6 wt % poly(methyl methacrylate) (PMMA) solution was obtained dissolving PMMA (MicroChem, 950.000 MW) in anisole. The solution was stirred for 1 h in a water bath at 75 °C. A protective PMMA film was obtained by spin-coating a drop of solution on the sample surface at 6 krpm for 30 s.

## Results and Discussion

### Growth, Morphology, and Structure

**Figure 1:**
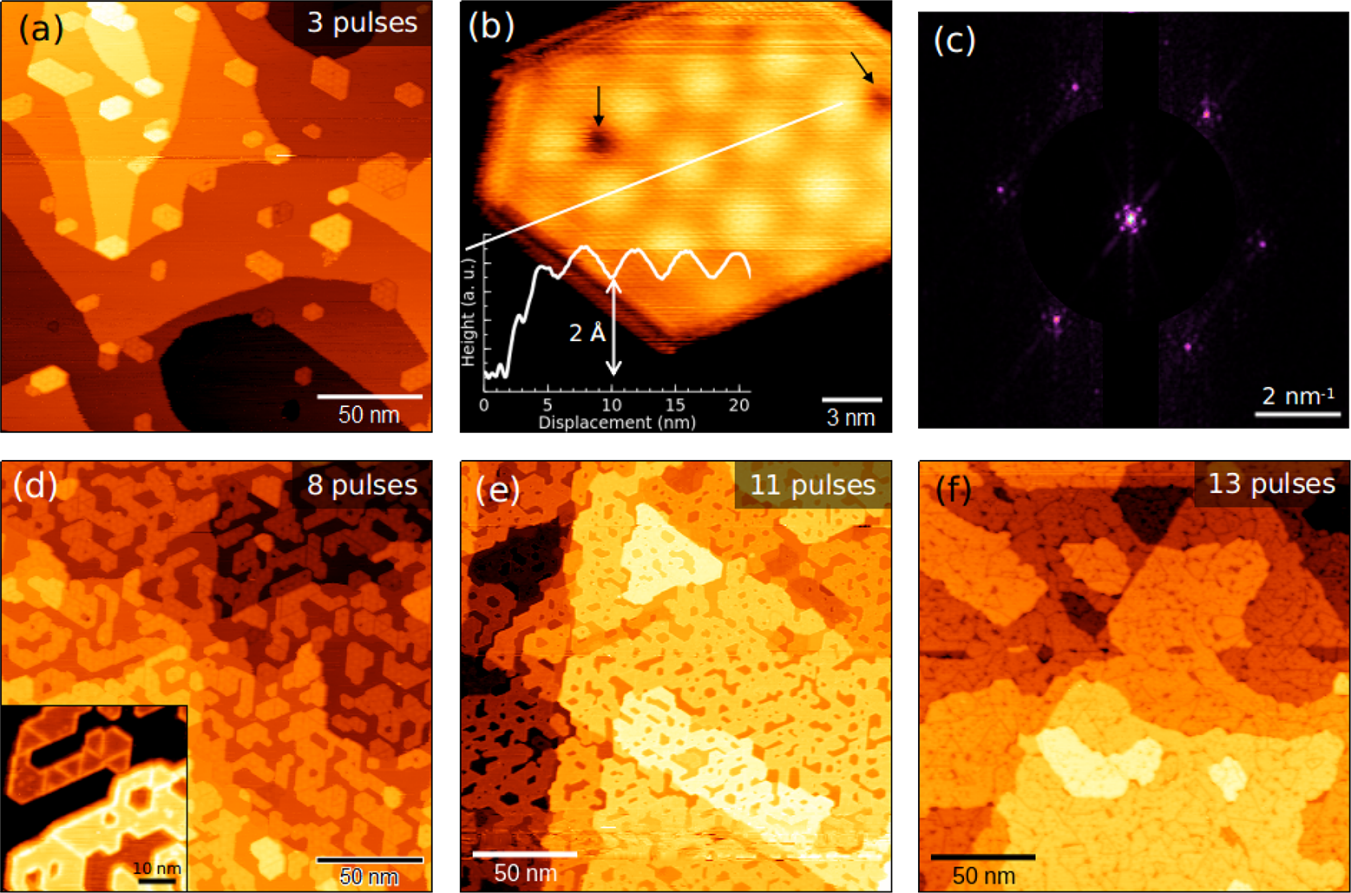
(a) Large-scale (200 × 200 nm^2^) STM image of SL MoS_2_ nanocrystals on Ag(111), grown by PLD using three laser pulses. (b) High-resolution STM image of a SL MoS_2_ nanocrystal. Black arrows indicate dark spots, presumably due to sulfur vacancies. Inset: line profile along the white line showing a 2 Å apparent height. (c) 2D Fourier Transform of the STM image in part b showing both MoS_2_ lattice and moiré spots. (d–f) 200 × 200 nm^2^ STM images of SL MoS_2_ on Ag(111) at different coverage. The three samples have been obtained by PLD with (d) 8, (e) 11, and (f) 13 laser pulses. (d) Inset: STM image showing mirror boundaries between nanocrystals (bias voltage, −1.85 V; set-point current, 0.4 nA).

In the PLD process, the amount of deposited MoS_2_ can be controlled by tuning the number of laser pulses which ablates the MoS_2_ target. We started from a low number of pulses to study the first growth stages on Ag(111). With three laser pulses (Figure 1a) and after annealing at 730 K in UHV, we observe the formation of 2D hexagonal shaped MoS_2_ nanoislands dispersed on the Ag surface. Most MoS_2_ nanocrystals are attached to Ag step edges, suggesting that monatomic steps provide preferential nucleation sites to MoS_2_ growth. This mechanism is likely to limit the step mobility, leading to a more disordered step arrangement with respect to pristine Ag(111) (figure S1a). Figure 1b shows a high-resolution STM image of a MoS_2_ nanocrystal. The measured apparent height is ∼2 Å (see inset), which agrees with the STM thickness of a single MoS_2_ layer grown on Au(111).^14,15^ The surface shows a hexagonal moiré pattern with ∼3.2 nm periodicity, due to the lattice mismatch between MoS_2_ and Ag(111). Fourier transforms of atomic resolution images (Figure 1c) show that moiré and MoS_2_ lattices are aligned, implying a negligible rotational mismatch between Ag(111) and MoS_2_. From the analysis of the moiré pattern,^16^ we obtain a MoS_2_ lattice parameter of 3.17 ± 0.02 Å, about 10% larger than the Ag(111) value, i.e. 2.89 Å. The measured lattice constant is close to the relaxed value of bulk MoS_2_, i.e. 3.16 Å.^17^ However, the finite uncertainty in room-temperature STM measurements, albeit relatively small, does not allow us to exclude a possible residual strain in MoS_2_ lattice (up to 1%), which may affect the vibrational properties, as discussed below. Dark spots (as those indicated by black arrows in Figure 1b) are normally observed on MoS_2_ surface. Comparably to MoS_2_/Au(111),^18^ these features could be related to sulfur vacancies, the most common point defect in MoS_2_, which are normally promoted by UHV annealing.

Having synthesized and observed isolated SL MoS_2_ nanocrystals on Ag(111), we aimed at increasing the MoS_2_ coverage to obtain a continuous SL film. To this purpose, we gradually increased the number of laser pulses: in the bottom panel of Figure 1, we report large-scale STM images acquired on three different samples obtained with 8 (d), 11 (e), and 13 (f) laser pulses. As coverage increases, the nanocrystals merge together into a connected SL structure, which gradually forms a uniform film on the Ag substrate (Figure 1e,f). The MoS_2_ lattice can grow on Ag(111) in two different orientations, rotated by 60° with each other. When differently oriented crystals merge together, they form mirror boundaries, which can be distinguished in STM images as straight lines between adjacent nanocrystals (Figure 1d, inset, at negative bias voltages the STM contrast of borders and mirror boundaries is usually enhanced). Therefore, the SL film is nanocrystalline, with mirror grain boundaries separating nanosized domains. Second layer islands start growing only after the first layer is completed (Figure S1b), suggesting a layer-by-layer growth mode driven by a strong film–substrate interaction (analogous to the PLD growth of SL MoS_2_ on Au(111)^15^). Since we focused on the study of SL MoS_2_, we did not increase the coverage any further to avoid the presence of a significant fraction of second layer.

### Stability under Ambient Conditions

**Figure 2:**
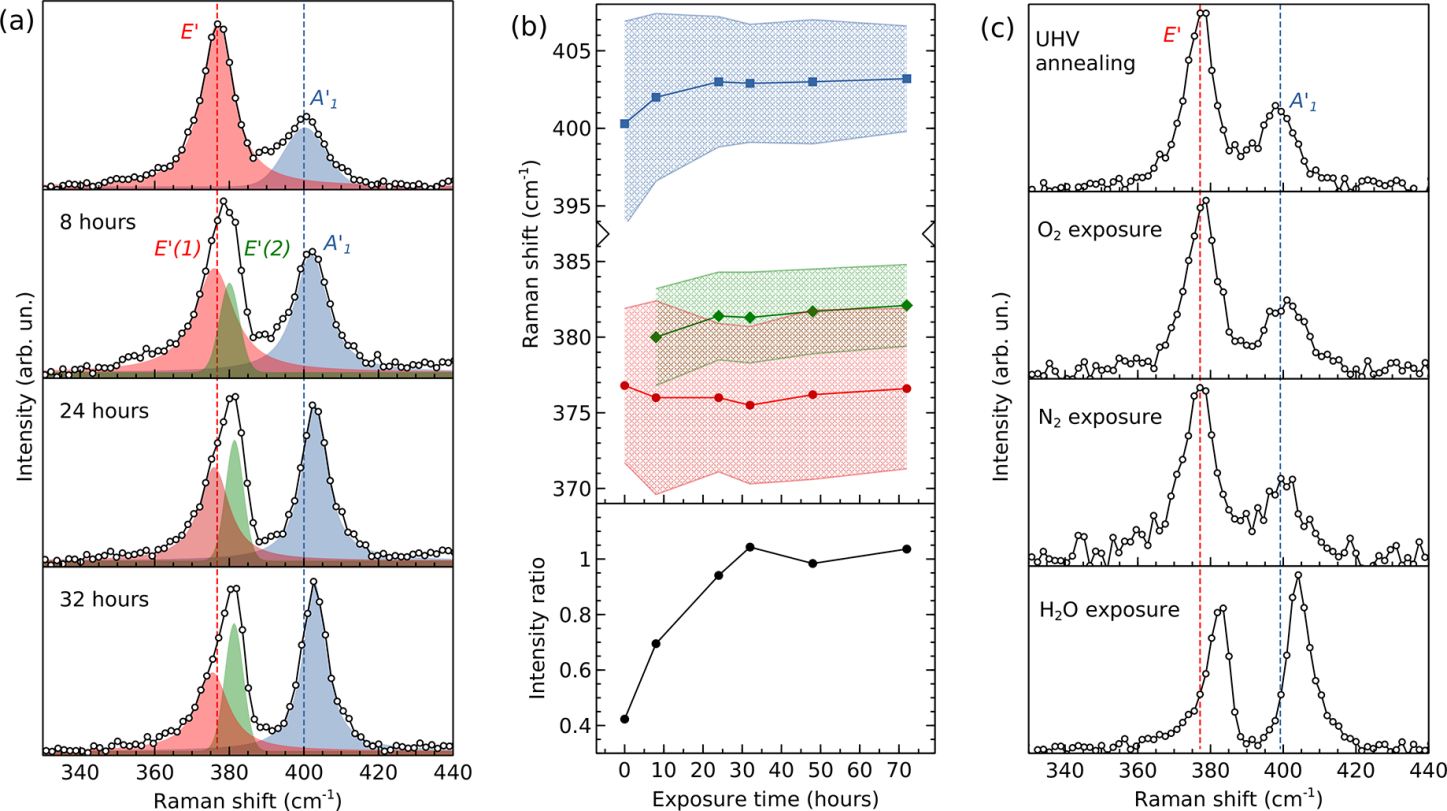
(a) Raman spectra of SL MoS_2_/Ag(111) acquired right after the sample was taken out from UHV (top), and after 8 (middle-top), 24 (middle-bottom) and 32 (bottom) h of exposure to ambient conditions. Red, green and blue curves are Voigt functions fitting the Raman peaks. Vertical dashed lines indicate the peak positions of E′ (red) and A_1_^′^ (blue). (b) Top: evolution of E′(1) (red), E′(2) (green) and A_1_^′^ (blue) peak positions and widths over exposure time. Color-shaded areas represent the fwhm of the Voigt components. Bottom: intensity ratio of the high-frequency over low-frequency peak (A_1_^′^/E^′^), reported as a function of air exposure time. (c) Raman spectra of SL MoS_2_/Ag(111) taken (top) after UHV annealing (600 K for 2 h) on a sample previously aged in air, and after 48 h exposure to O_2_ (middle-top), N_2_ (middle-bottom), and H_2_O (bottom). Vertical dashed lines indicate the peak positions of E′ (red) and A_1_^′^ (blue).

The SL MoS_2_ film on Ag(111) was then investigated ex situ by Raman spectroscopy, and constantly monitored to observe possible effects induced by air exposure over time. In the top panel of Figure 2a we report the Raman spectrum (457 nm excitation) obtained as soon as the sample was taken out from the UHV chamber. The plot shows the two main vibrational modes of SL MoS_2_, namely the in-plane mode E′ at 376.8 cm^–1^ and the out-of-plane A_1_^′^ at 400.3 cm^–1^. In the well-known spectrum of SL MoS_2_ on SiO_2_ (exfoliated or CVD-grown), the frequency difference between E′ and A_1_^′^ is about 18–20 cm^–1^ and the two modes are found at ∼384 and ∼403 cm^–1^, respectively.^19^ The ratio of A_1_^′^ over E′ intensity is ≥1 for a broad range of excitation wavelengths.^20^ In comparison, SL MoS_2_/Ag(111) shows a downshift of both modes and a much lower A_1_^′^/E^′^ intensity ratio of ∼0.4. Previous works have shown that strain^21^ and doping^22^ have considerable impact on MoS_2_ Raman features. In-plane biaxial strain mainly influences E′, which downshifts at a rate of ∼5 cm^–1^ per 1% of tensile strain, while ∼2 cm^–1^/% is the downshift rate for A_1_^′^.^7,21^ Using these values and the measured shift of E′ and A_1_^′^, we can infer a 1.4% in-plane biaxial tensile strain, which is approximately compatible with our STM measurements. However, the observed shifts can be further contributed by other effects, besides in-plane strain. For instance, the Ag(111) substrate may induce out-of-plane strain due to the interaction with contact S atoms, and n-type doping,^23^ which is known to soften, broaden, and dampen the A_1_^′^ mode.^22^ Both these mechanisms, concurrently with in-plane strain, can contribute to the anomalous Raman response observed on MoS_2_/Ag(111). Also, the strong photoluminescence signal associated with the direct gap of SL MoS_2_ (e.g., detected on MoS_2_ exfoliated on SiO_2_^24^) is totally quenched on Ag(111) (Figure S2), likely due to electron–hole separation favored by the metal contact.

After ∼8 h in ambient conditions, we measured again the Raman spectrum (Figure 2a, 8 h panel). The low-frequency feature is now contributed by two peaks, referred to as E′(1) (red) and E′(2) (green), whose coexistence is discussed below. The A_1_^′^ peak upshifts and its relative intensity increases. For increasing air exposure time (Figure 2a, 24 and 32 h panels), E′(2) and A_1_^′^ become more intense with respect to E′(1), and upshift to ∼382 and ∼403 cm^–1^, respectively (i.e., toward typical positions of SL MoS_2_ on SiO_2_), while the frequency of E′(1) is essentially unvaried at 376–377 cm^–1^. The observed behavior of MoS_2_/Ag(111) Raman modes is reported in Figure 2b: the top panel shows E′(1), E′(2), and A_1_^′^ frequencies and line widths as a function of air exposure time, while the bottom panel shows the measured ratio of A_1_^′^ intensity over the peak intensity (i.e., sum of E′(1) and E′(2)) of the in-plane vibration. The most significant variations are observed within 48 h of air exposure, after which an equilibrium situation is reached with the main peaks found at 382.5 and 404 cm^–1^, and the A_1_^′^/E^′^ intensity ratio slightly above 1. At equilibrium, no significant changes are observed over 1 month (Figure S3). The upshift of vibrational frequencies and the increase in intensity ratio suggest that the exposure to ambient conditions weakens the interaction between SL MoS_2_ and Ag, responsible for the anomalous Raman response observed on a pristine sample (Figure 2a, top panel). In this picture, the coexistence of the two contributions, E′(1) and E′(2), to the in-plane vibration can be attributed to the simultaneous sampling of two distinct types of regions within the laser spot (diameter of ∼2 μm on the sample surface): a region where MoS_2_ strongly interacts with Ag and the other where such interaction is weaker. Thus, the gradual decrease of E′(1) against E′(2) over time suggests that the region of weak interaction becomes predominant over the other. Despite the evolution of vibrational properties, the PL signal is always totally quenched (figure S2), suggesting that a channel for charge separation or nonradiative decay is still active.

Aiming at restoring the original MoS_2_–Ag interaction, we put the sample back in UHV and annealed it for 2 h at 600 K. Once out the UHV system, its Raman spectrum (Figure 2c, top) essentially overlaps with the pristine spectrum (Figure 2a, top). This result proves that a mild annealing in UHV restores the condition prior to the short-term aging induced by air exposure. To more deeply investigate the aging mechanism, we exposed the sample for 48 h to controlled O_2_ (1 bar), N_2_ (1 bar), and H_2_O (volume corresponding to 75% relative humidity; see Methods for further details) atmospheres, with the aim to possibly discriminate the different contributions of air components. The Raman spectra acquired ex situ immediately after each exposure step are shown in Figure 2c and labeled accordingly. O_2_ and N_2_ exposures do not induce any significant difference in the Raman spectrum of SL MoS_2_/Ag(111), whereas exposure to H_2_O vapor results in the same variation of Raman modes discussed before. Our experiments thus point at humidity as the main cause for the short-term aging of SL MoS_2_/Ag(111) in ambient conditions. Since the behavior of Raman modes can be associated with the weakening of the MoS_2_–Ag interaction, we argue that H_2_O molecules gradually intercalate at the MoS_2_/Ag interface, thus lifting MoS_2_ up from the metallic substrate. The intercalated regions increase over time, leading to the observed evolution of Raman features. Water intercalation caused by air exposure has been reported for SL MoS_2_ on hydrophilic dielectric substrates, e.g., Al_2_O_3_.^25^ In our case, the intercalation could be favored by the hydrophilic character of Ag(111).^26^ Interestingly, we do not observe any aging effects on SL MoS_2_ grown on Au(111) using the same PLD method. Figure S4 shows the Raman spectra of MoS_2_/Au(111) as a function of air exposure time: the as-exposed spectrum (acquired as soon as the sample was taken out from UHV) is similar to the pristine MoS_2_/Ag(111) spectrum (Figure 2a, top), but in contrast to MoS_2_/Ag(111), no variations are observed for increasing exposure time. The different behavior of SL MoS_2_/Au(111) can be due to the low hydrophilicity of Au, which has lower water adsorption energy and wettability with respect to other metal surfaces.^27−29^ This substrate effect corroborates the hypothesis that the aging of MoS_2_/Ag(111) is due to water intercalation, rather than, e.g., water adsorption at the MoS_2_ surface. The intercalation could be locally favored by the presence of defects, such as grain boundaries and sulfur vacancies (see Figure 1b–d), which are known to enhance the local reactivity of TMDs in ambient conditions.^30^

**Figure 3:**
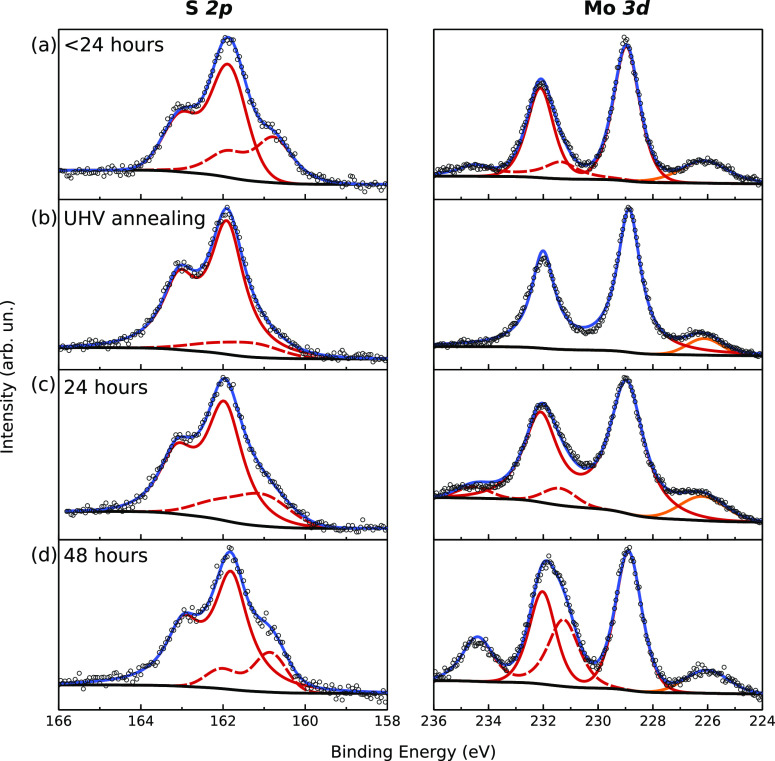
S 2p (left) and Mo 3d (right) core levels, where open circles are raw data, blue curve is the full fit after background removal (black curve), orange curve is S 2s core level, red solid curves are Mo^4+^ and S^2–^ states for MoS_2_, and red dashed curves are additional states discussed in the main text, obtained: (a) after less than 24 h air exposure, (b) after annealing in UHV for 3 h at 600 K, (c) after 24 h air exposure, and (d) after 48 h air exposure (upon second annealing as in part b).

XPS was carried out to study the chemical stability of MoS_2_ on Ag(111). Figure 3a shows the S 2p (left) and Mo 3d (right) core levels after air exposure (less than 24 h). The Mo 3d spectrum is nearly overlapped with the S 2s core level (orange line). The main doublet (red line) at binding energy (BE) 228.97 eV is related to the Mo^4+^ ion of MoS_2_ and is in good agreement with the bulk reference (Figure S5). The smaller doublet (red dashed curve) at 231.36 eV can be attributed to substoichiometric molybdenum oxysulfide (MoO_*x*_S_*y*_) because typically MoO_3_ is found at higher BE (232.7 eV).^31^ More interestingly even S 2p is composed of two distinct doublets. The former (red curve) at 161.87 eV is related to S^2–^ state of MoS_2_ (again in good agreement with bulk, Figure S5), whereas the latter (red dashed) at lower BE (160.76 eV) deserves a deeper understanding and will be discussed in the following. After annealing the sample in UHV at 600 K for 3 h (Figure 3b), the low-BE S 2p doublet strongly decreases and shifts at higher BE, while the smaller MoO_*x*_S_*y*_ doublet in the Mo 3d spectrum disappears. Therefore, the emergence of both these features is related to air exposure and can be reversed by UHV annealing. In principle, the low-BE S 2p doublet could be associated with the possible formation of Ag_2_S^32^ alloy, sulfur vacancies^33^ or Mo–oxysulfides.^34^ The hypothetical contribution of Ag_2_S can be ruled out by the following evidence. First, the BE difference between the two S 2p components is about 1 eV and such a difference is not observed in the Ag 3d core level, whose BE and line shape are unaffected by UHV annealing or air exposure (data not shown). Second, the observed exposure/annealing behavior would lead to the counterintuitive conclusion that Ag_2_S alloying results from air exposure and is reversed by UHV annealing. The latter argument allows us to rule out also the possible contribution of S vacancies, whose formation/removal could be hardly correlated to the exposure/annealing cycle. Therefore, we attribute the low-BE S 2p component to the formation of Mo–oxysulfide compounds,^34^ also responsible for the high-BE doublet of Mo 3d, as pointed out before.

The annealed sample is then exposed to air for 24 h (Figure 3c), thus recovering the Mo 3d and S 2p spectra of Figure 3a. The same sample is then annealed again at the same temperature (600 K) and subsequently exposed for 48 h (Figure 3d). The low-BE S 2p doublet is fully restored (as in Figure 3a) and the high-BE of the Mo 3d line noticeably increases. Comparing the O 1s spectra after 24 and 48 h (Figure S6), we observe an increase of a high-BE component typically related to hydroxyl groups (OH), compatible with reported MoO_*y*_S_*z*_ compound with oxygen-rich composition.^34^ The gradual formation of a MoO_*x*_S_*y*_ phase, mediated by reactive hydroxyl groups, further confirms the time-dependent interaction between water vapor and MoS_2_/Ag. The possibility to restore the original condition by UHV annealing suggests that hydroxyl groups are weakly bounded and can be removed to recover the pristine MoS_2_–Ag interface.

To prevent sample aging, we adopted the following strategy aiming at hindering water intercalation at the MoS_2_–Ag interface. After annealing the sample in UHV for 2 h to restore the MoS_2_–Ag interaction (confirmed by means of Raman investigation), we spin-coated PMMA on the sample surface obtaining a capping film. At variance with the analysis reported in Figure 2a, the Raman spectra of the PMMA-capped MoS_2_ turn out to be unaffected by the environmental humidity even after one month of ambient condition exposure (Figure S7). More in detail, in terms of time evolution, the characteristic E′ and A_1_^′^ Raman modes show neither the frequency upshift nor the relative intensity switch exhibited by the uncapped sample. As a matter of fact, the A_1_^′^/E^′^ intensity ratio is constantly below 1 throughout the considered temporal window. We concluded that the PMMA capping layer is effective in creating a barrier against water intercalation, thus preserving the strong MoS_2_–Ag interaction, observed in the pristine sample, on a time scale of months.

## Conclusions

We synthesized SL MoS_2_ on Ag(111) by PLD, observed its structure by in situ STM and studied its stability by Raman spectroscopy and XPS. The moiré pattern observed on pristine MoS_2_/Ag(111) is indicative of the high-purity interface obtained with the employed UHV-PLD scheme, a condition proved to be relevant for the fabrication of low-resistance contacts.^35^ The strong interaction with the metallic substrate has profound effects in MoS_2_ Raman modes, whose frequencies and intensities are affected by strain and doping induced by the Ag substrate. Air exposure affects the chemical stability of SL MoS_2_/Ag(111) over a time-scale of a few hours. The main aging mechanism is identified in water intercalation at the MoS_2_/Ag interface, causing the formation of Mo–oxysulfides, which can be reversed by UHV annealing. A PMMA capping layer, applied immediately after air exposure, efficiently protects the sample from water, preventing the related aging. Our findings clearly show the influence of the Ag substrate on the properties of SL MoS_2_, along with the importance of interface effects in the heterostructure stability. This work deepens our understanding of TMD/metal systems providing relevant insight into their interface physics, which plays a pivotal role in the performances of TMD devices.

## Supporting Information Available

Additional STM, Raman, photoluminescence and XPS data (PDF)
