# Supplementary material for: Hydrophilic Character of Single-Layer MoS2 Grown on Ag(111)
Source: J Phys Chem C Nanomater Interfaces. 2021 Apr 27;125(17):9479–85. doi: 10.1021/acs.jpcc.1c01768 (PMC8154856; doi:10.1021/acs.jpcc.1c01768)
Supplement: Supplementary file 1 — jp1c01768_si_001.pdf [file jp1c01768_si_001.pdf]

SUPPLEMENTARY INFORMATION

Hydrophilic Character of Single-Layer MoS_2 Grown on Ag(111)

Francesco Tumino[1,*], Carlo Grazianetti[2,**], Christian Martella[2], Marina Ruggeri[1], Valeria Russo[1], Andrea Li Bassi[1], Alessandro Molle[2] and Carlo S. Casari[1]

[1]Department of Energy, Politecnico di Milano, via G. Ponzio 34/3, Milano, I-20133, Italy
[2]CNR-IMM Unit of Agrate Brianza, via C. Olivetti 2, Agrate Brianza, I-20864, Italy

E-mail: * francesco.tumino@polimi.it, ** cgrazianetti@mdm.imm.cnr.it

Supplementary data

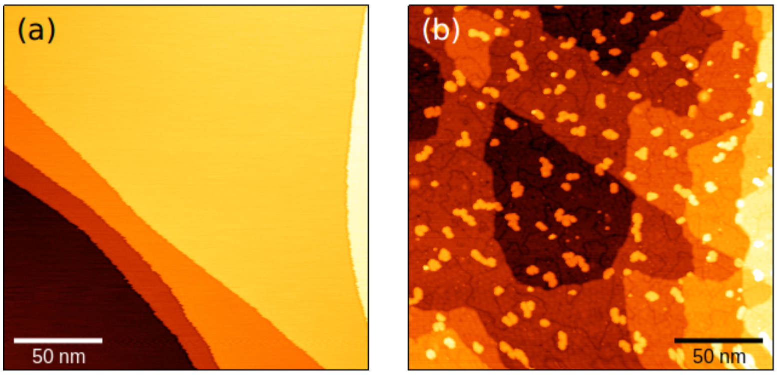

Figure S1: (a) STM image of pristine Ag(111). (b) STM image of MoS_2 on Ag(111) produced by PLD with 15 laser pulses. The bright clusters are second layer islands growing on top of the single-layer film.

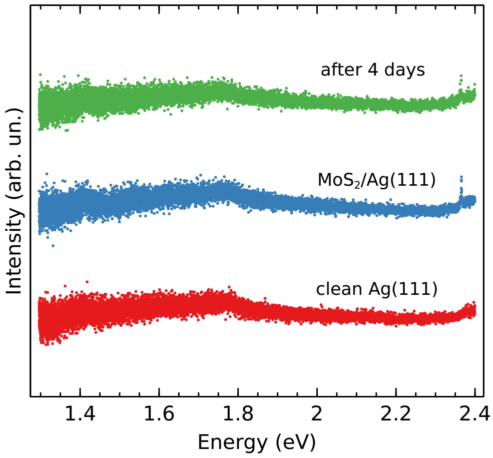

Figure S2: Photoluminescence spectra of clean Ag(111) (red), freshly exposed MoS_2/Ag(111) (blue) and after 4 days in ambient conditions. The data are vertically stacked for clarity.

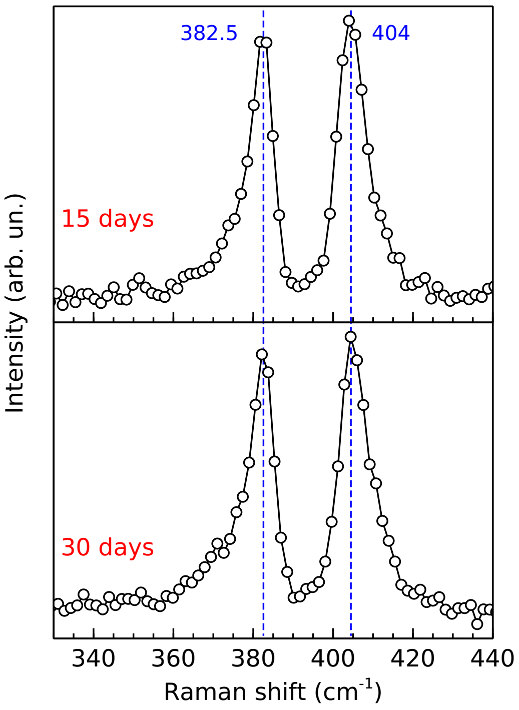

Figure S3: Raman spectra of SL MoS_2 on Ag(111) acquired after 15 days (top) and 30 days (bottom) of air exposure. Blue dashed lines indicate the main peaks positions at 382.5 and 404 cm^{-1}.

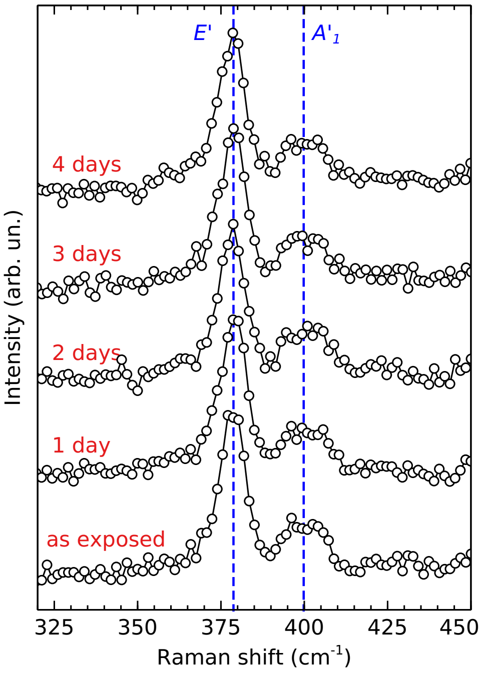

Figure S4: Raman spectra of single-layer MoS_2 on Au(111) for increasing air exposure time. The spectra are vertically stacked for clarity. Blue dashed lines indicate the peak positions of E' and A'_1.

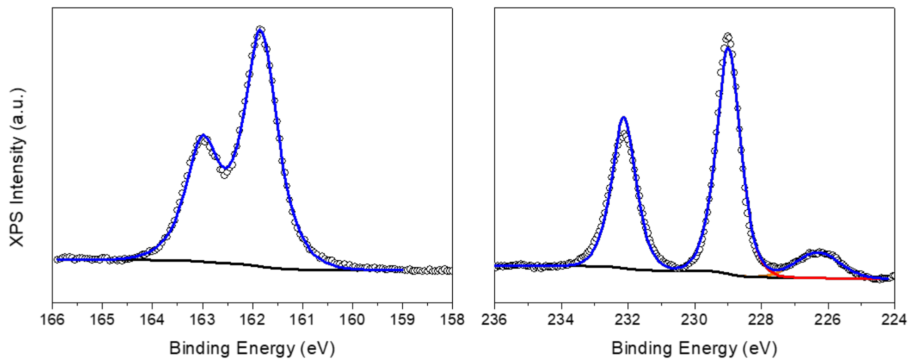

Figure S5: S 2p (left) and Mo 3d (right) core levels of reference bulk MoS_2 crystal. Open circles are raw data, blue curve is the full fit after background removal (black curve), orange curve is S 2s core level, red solid curves are Mo^{4+} and S^{2-} states.

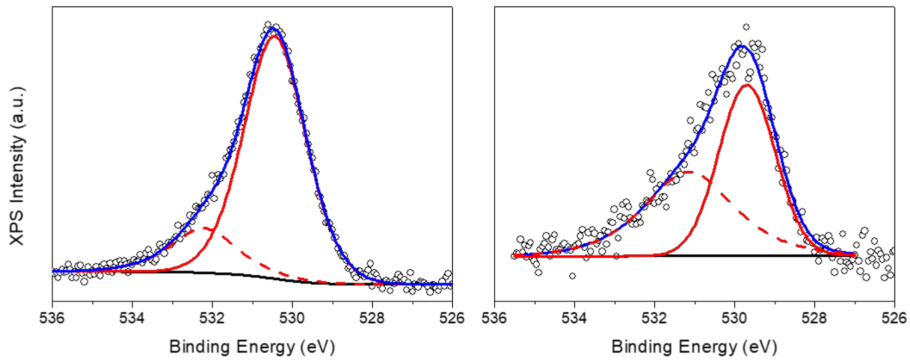

Figure S6: O 1*s* core level after 24 hours air exposure (left) and after 48 hours (right). Red solid curves are related to adsorbed O_2, red dashed curves to OH-groups, blue solid curves are full fit of raw data reported as open circles.

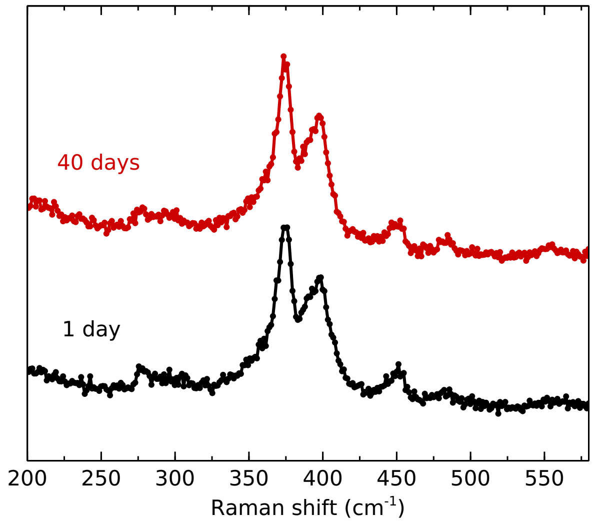

Figure S7: Comparison of the Raman spectra after one day (black curve) and forty days (red curve) after the capping with the PMMA protective layer.
